# Multi-focus image fusion dataset and algorithm test in real environment

**DOI:** 10.3389/fnbot.2022.1024742

**Published:** 2022-10-18

**Authors:** Shuaiqi Liu, Weijian Peng, Wenjing Jiang, Yang Yang, Jie Zhao, Yonggang Su

**Affiliations:** ^1^College of Electronic and Information Engineering, Hebei University, Baoding, China; ^2^Machine Vision Technological Innovation Center of Hebei, Baoding, China; ^3^National Laboratory of Pattern Recognition (NLPR), Institute of Automation, Chinese Academy of Sciences, Beijing, China

**Keywords:** image fusion, multi-focus image fusion dataset, image preprocessing, multi-focus image fusion algorithm test, real environment

## Introduction

In the past three decades, not only some classical MIF datasets have appeared, but also MIF technology has developed rapidly (Zheng et al., 2020; Zhu et al., 2021). The existing MIF datasets can be divided into two categories, namely, the simulated image dataset obtained by applying Gaussian blur to the existing image dataset and the benchmark image dataset captured by the professional camera. The source image after Gaussian blurring in the multi-focus simulated image dataset are difficult to reflect the information of focused and unfocused objects in the real environment. The benchmark image dataset also has imaging equipment limited to professional cameras. Both of them are difficult to achieve the application of MIF technology in the real environment.

MIF algorithms can be classified into three categories i.e., spatial domain fusion algorithms, transform domain fusion algorithms, and fusion algorithms based on deep learning (Liu et al., 2021). The spatial domain fusion algorithms mainly take pixel-level gradient information or image blocks for fusion. Bouzos et al. (2019) presented a MIF algorithm based on conditional random field optimization. Xiao et al. (2020) presented a MIF algorithm based on Hessian matrix. The transform domain fusion algorithms consist of three processes: image transformation, coefficient fusion and inverse transformation. Liu et al. (2019) proposed a MIF algorithm based on an adaptive dual-channel impulse cortical model and differential images in non-subsampled Shearlet transform (NSST) domain. In recent years, the fusion algorithms based on deep learning have become a research hotspot in the field of multi-focused image fusion. Zhang et al. (2020) proposed an image fusion framework based on convolutional neural network, which utilizes two convolutional layers to extract salient features from source images. Liu et al. (2022) proposed a MIF algorithm based on low vision image reconstruction and focus feature extraction. Although these MIF algorithms have achieved good image fusion results among these public datasets, the image fusion databases used by these algorithms are all data taken by professional cameras or synthetic data, which cannot reflect the fusion performance of the fusion algorithm in the real environment.

As mentioned above, in the past few years, a series of MIF algorithms have been developed by scholars from various countries. To test the performance of these algorithms, some classic public MIF datasets have occurred. Currently, the commonly used datasets include Multi Focus-Photography Contest dataset (http://www.pxleyes.com/photography-contest/19726), Lytro color multi-focus image dataset (Nejati et al., 2015), Savic dataset (http://dsp.etfbl.net/mif/) and Aymaz dataset (https://github.com/sametymaz/Multi-focus-Image-Fusion-Dataset), etc. Some of these datasets were captured by professional cameras, and others were obtained by applying Gaussian blur to existing image datasets. The Multi Focus-Photography Contest dataset is an image photography competition held by the Photography Contest website. It contains 27 pairs of multi-focus images. Images in Lytro multi-focus dataset were acquired by the Lytro camera which is an all-optical camera whose imaging system employs a microlens array focused on the focal plane of the camera's main lens. The Lytro multi-focus dataset includes 20 groups of color multi-focus images and four sets of multi-source focus images. The image resolution is and the image format is jpg. The Savic dataset is collected by Nikon D5000 camera and contains 27 pairs of images. In Savic dataset, 21 pairs of images with format jpg are taken indoors, and 6 pairs of images with format bmp are used for MIF algorithm testing. In Aymaz dataset, the 150 multi-focus images are obtained by using the Gaussian blur function to locally blur some common image datasets. This dataset also contains some multiple source images of the same scene with different focal points. In addition to color multi-focus datasets, there are also some grayscale multi-focus datasets, and some images in grayscale multi-focus datasets.

The above-mentioned datasets can well reflect the performance of the fusion algorithms to some extent. However, these datasets can hardly reflect the application of MIF techniques in real environment. At present, the most commonly used camera device in daily life is the smartphone. With the continuous development of the imaging technology, the smartphone photography is more and more recognized by people. Therefore, it is necessary to try to construct a real-environment dataset by using different smartphones. In order to better build the database and collect images of the real environment more widely, we selected five mobile phones that were among the top ten in sales nationwide at that time for data collection such as HUAWEI Mate 30, OPPO Reno Z, Honor30 Pro+, Honor V30 Pro and iPhone XR to collect the multi-focus images in HBU-CVMDSP dataset. There are some unavoidable problems in collecting images with mobile phones, such as jitter, not completely overlapped and brightness. To address these issues, the proposed dataset is pre-processed after acquisition with image cropping, standardization of basic image attributes and image alignment. The contributions of this paper are as follows: In this paper, we construct a real-environment dataset named as HBU-CVMDSP, which includes 66 groups of multi-focus images. we give the detail of how to pre-process the raw data of the real-environment dataset, and the experiments prove that it is effectively for testing the fusion algorithms. We also test the performance of some existing image fusion algorithms on the HBU-CVMDSP dataset.

## Collection and construction of the dataset

Due to the variability of image effects from different smartphones, five different models of smartphones shown in Table 1 are used for image collection in this paper.

**Table 1:** Acquisition equipment.

**Smartphone model**	**Camera description**
HUAWEI Mate 30	Rear triple camera layout: 40-megapixel (MP) camera, 16 MP super-wide-angle camera and 8 MP telephoto camera
OPPO Reno Z	Rear dual-camera layout: 48 MP camera and 5 MP depth-of-field lens
Honor 30 Pro+	Rear three-camera layout: 50 MP super-sensitive camera, 16 MP super-wide-angle camera and 8 MP telephoto camera
Honor V30 Pro	Rear triple camera layout: 40 MP main camera, 12 MP super-wide-angle camera and 8 MP telephoto camera
iPhone XR	Rear single-camera layout: 12 MP wide-angle camera

**Figure 1:**
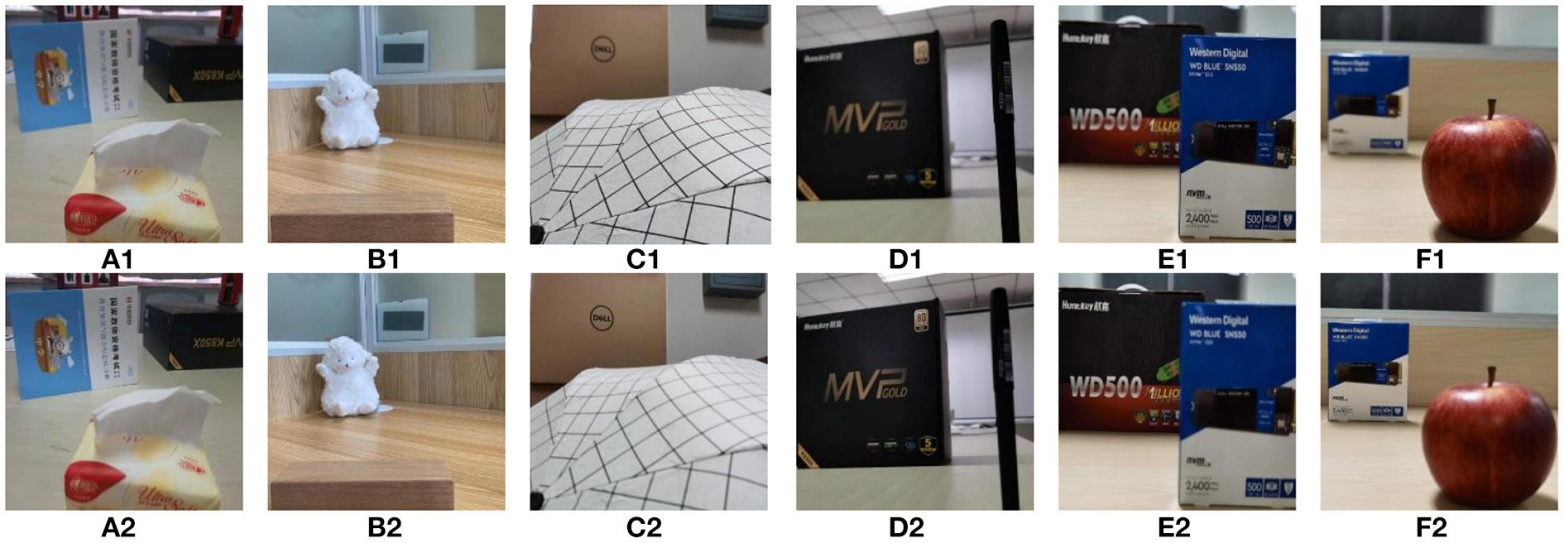
Some images selected from the HBU-CVMDSP dataset. **(A1–F1)** are the foreground focused image in a group of multi focus images. **(A2–F2)** are the background focused image in a group of multi focus images.

In this paper, the constructed real-environment multi-focus image dataset is named as HBU-CVMDSP. There are two kinds of sceneries i.e., natural scenery and artificial scenery in HBU-CVMDSP dataset, and these sceneries are selected from the laboratory, campus, gymnasium, and shopping mall, respectively. The HBU-CVMDSP dataset contains 66 groups of multi-focus images with jpg format. The image size is uniformly cropped to 512 × 512 to ensure the efficient execution of the experiment. Figure 1 shows some images in HBU-CVMDSP dataset.

## Image preprocessing

**Figure 2:**
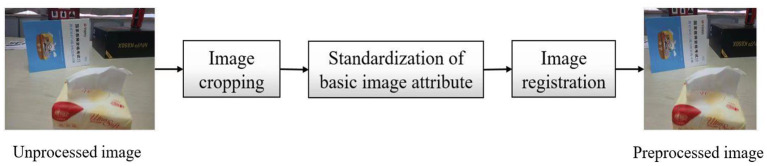
Schematic diagram of Image preprocessing.

In order to solve these unavoidable problems when capturing images with mobile phones, the proposed dataset is preprocessed by image clipping, standardization of basic image attributes and image registration after acquisition, such as Figure 2.

To further illustrate the necessity of image preprocessing, before the dataset is preprocessed, we use dense scale-invariant feature transform (DSIFT) (Liu et al., 2015) and CNN (Liu et al., 2017) based image fusion algorithms to examine the dataset. The partial fusion results of the DSIFT and CNN can be found in https://www.researchgate.net/publication/359468841.

It can be seen from the above results that the fusion effects are not visually satisfactory. The ghosting at the image edges is mainly due to the misregistration of the images in the dataset, while the blocking and distortion in the images are due to the inconsistency of the brightness between the two source images in the dataset. Therefore, we conduct image cropping, standardization of basic attributes and registration processing on the dataset to ameliorate the quality of the fused images. If the mobile phone device shoots scenes with different focus areas, the obtained image field of view will be different. When the image background information is clear, the field of view is wider, and when the image near field information is clear, the view is narrower. Therefore, if two images with different focal points have the same size, the field of view of the two images will be different, and the ghosting will appear during the fusion process. In addition, the slight jitter when taking pictures will also lead to a slight gap in the field of view of two images. The images in HBU-CVMDSP dataset are cropped using the nearest neighbor interpolation algorithm. The details be found in https://www.researchgate.net/publication/359468841.

When smartphones collect a foreground and background focused image, due to the different depth of field, the attributes such as brightness and contrast of the image will be different. A group of images with different attributes will affect the matching of feature points in the image registration process, and the fusion image will appear block effect, resulting in unsatisfactory fusion result. In this paper, we standardize the basic attributes of color images using the SHINE_color toolbox (Willenbockel et al., 2010). When standardizing the basic attributes of images, we designate one image in the image group as the source image and the other image as the target image. Firstly, the source image and target image are transformed from RGB space to HSV space. Then the chroma, saturation and luminance are separated, the standardization of the basic attributes of the images is accomplished by adjusting the luminance channel of the source image and the target image to be equal in spatial frequency and direction. In this paper, the SIFT algorithm is used for image registration.

## Experimental results and analysis

### Experiment and analysis

In this experiment, we use the following nine metrics to quantitatively evaluate the performance of the image fusion algorithms: (1) Normalized mutual information (NMI), which can effectively improve the stability of the MI (Liu et al., 2020). (2) Nonlinear correlation information entropy (NCIE), which is a metric used to evaluate the quality of the fusion image (Su et al., 2022). (3) Gradient-based evaluation metric *Q*_*G*_ (Liu et al., 2020), which is used to evaluate the gradient information of the source image retained in the fused image. (4) Phase consistency based evaluation metric was proposed in Liu et al. (2020). (5) Structural similarity based evaluation metric *Q*_*S*_, which is an image quality evaluation metric based on the universal quality index (Liu et al., 2020). (6) Structural similarity based evaluation metric *Q*_*Y*_ (Liu et al., 2020). (7) Human perception based evaluation metric *Q*_*CB*_, which can be used to evaluate the contrast information between images (Liu et al., 2020). (8) Human perception based evaluation metric *Q*_*CV*_, which is an image fusion evaluation metric based on human visual perception (Liu et al., 2020). (9) Tsallis entropy is a generalization of Shannon entropy, which can be used to evaluate the retentive information between the source image and the fusion image. For *Q*_*MI*_, *Q*_*NCIE*_, *Q*_*G*_, *Q*_*P*_, *Q*_*S*_, *Q*_*Y*_, *Q*_*CB*_, and *Q*_*TE*_, the higher the value of them is, the better the fusion result will be. And for the *Q*_*CV*_, the smaller the value is, the better the fusion result will be.

#### Ablation experiment

To validate the importance of the pre-processing of the dataset, we use DSIFT and CNN fusion algorithms to conduct the fusion experiments on the dataset before and after image registration, and compare the subjective and objective fusion results of the two fusion algorithms. The experiments are completed by a PC with Intel core i5-10500, 3.10 GHz CPU, 8GB RAM memory, and NVIDIA GeForce GTX 1660 SUPER GPU. Due to space limitation, we only give the experimental results of the DSIFT algorithm. The experimental results of the CNN algorithm are shown in https://www.researchgate.net/publication/359468841.

**Figure 3:**
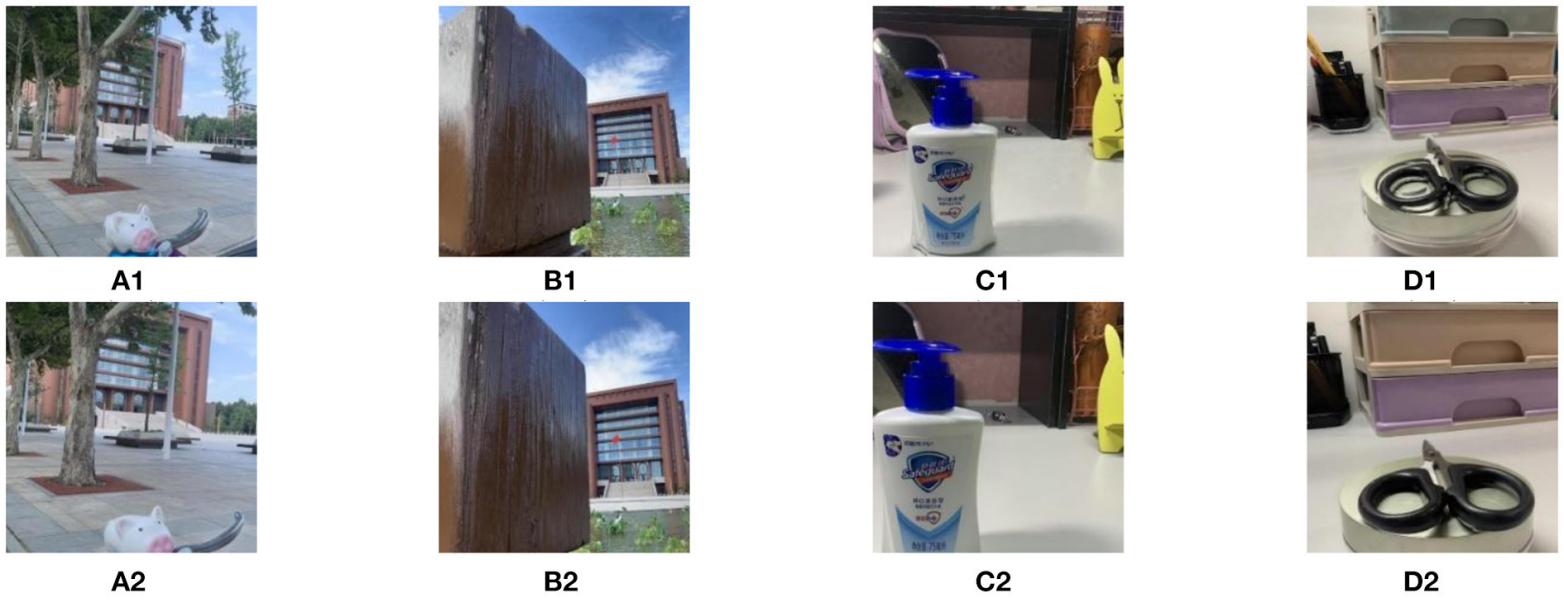
The fusion results before and after image registration. **(A1–D1)** are the fused images for the multi focus image pair without registration processing. **(A2–D2)** are the fused images by registration images.

The fusion results of the DSIFT algorithm are shown in Figure 3. The first row and second row in Figure 3 are the fusion results corresponding to the dataset before image registration and the dataset after image registration, respectively. Obviously, after image registration, the visual effects of the fused images in the second row have been significantly improved.

In addition, we calculate the values of *Q*_*MI*_, *Q*_*TE*_, *Q*_*NCIE*_, *Q*_*G*_, *Q*_*P*_, *Q*_*S*_, *Q*_*Y*_, *Q*_*CV*_, and *Q*_*CB*_ of the fused images obtained by DSIFT algorithm on the dataset before and after image registration, respectively. The values of the nine metrics are shown in Table 2, respectively, from which one can find that in addition to the decrease of the *Q*_*CV*_ value, the *Q*_*MI*_, *Q*_*TE*_, *Q*_*NCIE*_, *Q*_*G*_, *Q*_*P*_, *Q*_*S*_, *Q*_*Y*_, and *Q*_*CB*_ values of the fused images obtained by the DSIFT on the dataset after image registration are all increased. Therefore, conducting the image registration process on the dataset can effectively improve the performance of the fusion algorithms both in subjective vision and objective evaluation.

**Table 2:** The nine metrics' values of the fused images before and after image registration.

**Test image**	**Preprocessing**	***Q*_*MI*_**	***Q*_*TE*_**	***Q*_*NCIE*_**	***Q*_*G*_**	***Q*_*P*_**	***Q*_*S*_**	***Q*_*Y*_**	***Q*_*CV*_**	***Q*_*CB*_**
Piggy image	Before registration	1.029	0.3761	0.8445	0.6564	0.5465	0.854	0.9568	165.3	0.6904
	After registration	1.2	0.4389	0.8572	0.6861	0.6957	0.9339	0.9709	51.29	0.7578
Wood pile image	Before registration	1.021	0.3657	0.833	0.6702	0.6836	0.9043	0.9321	77.48	0.7011
	After registration	1.135	0.4046	0.8393	0.7185	0.7571	0.9478	0.9736	57.23	0.7788
Handwashing fluid	Before registration	1.192	0.4137	0.8474	0.6458	0.6512	0.9187	0.9105	145.9	0.6569
image	After registration	1.31	0.4443	0.8562	0.6771	0.7512	0.9659	0.9287	13.14	0.6974
Scissors image	Before registration	0.9387	0.3545	0.8279	0.6109	0.4073	0.8662	0.852	92.77	0.5971
	After registration	1.208	0.4276	0.8479	0.641	0.7335	0.9425	0.9208	7.673	0.6936

**Figure 4:**
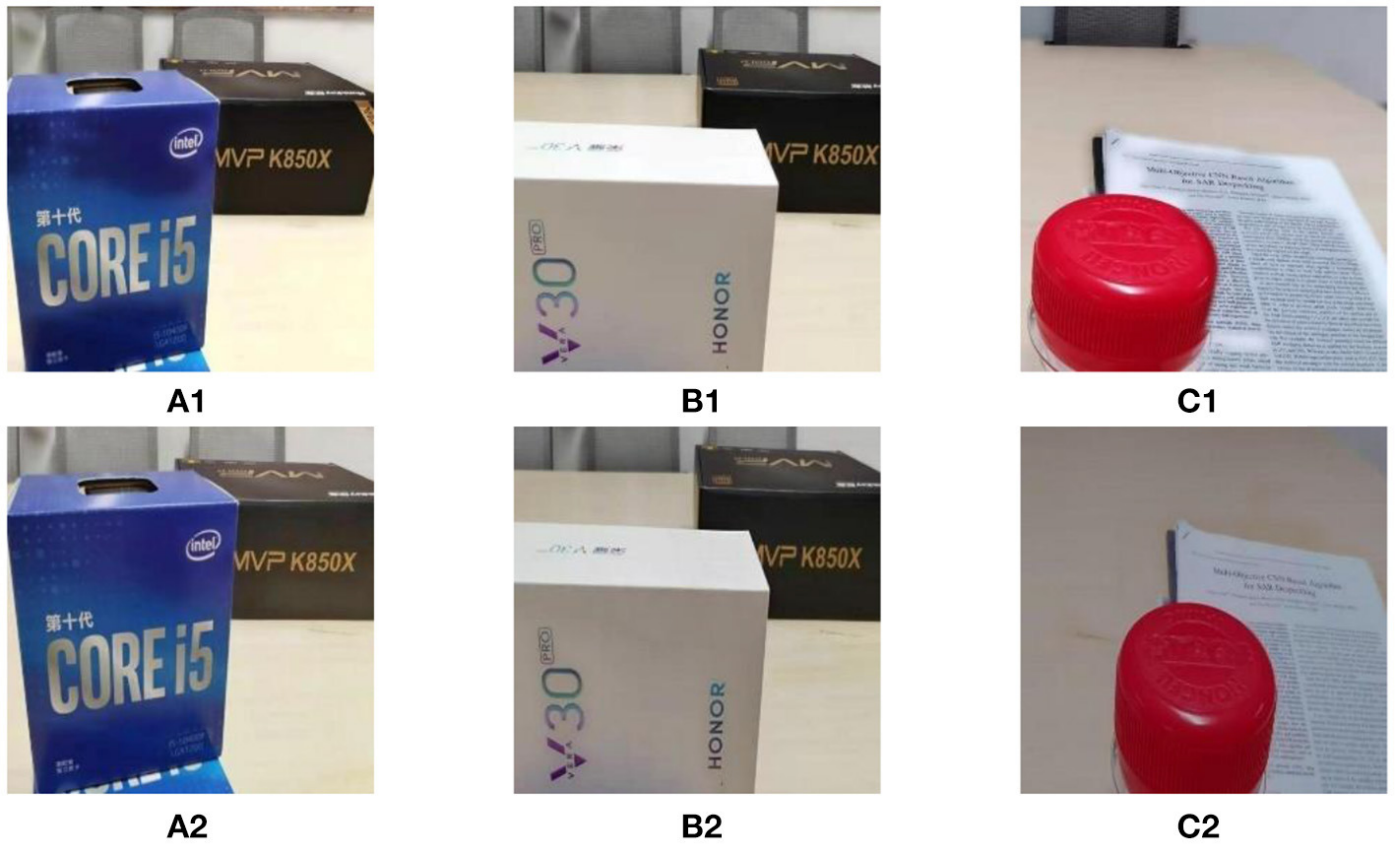
The fusion results before and after standardization of the image basic attribute. **(A1–C1)** are the fused images for the multi focus image pair without image basic attribute standardization processing. **(A2–C2)** are the fused images by image basic attribute standardization processing.

The fusion results of the DSIFT algorithm on the dataset before and after standardizing the basic attributes of images are shown in the first row and second row of the Figure 4, respectively. After the standardization of the image basic attribute, the visual effects of the fused images shown in the second row of the Figure 4 have been significantly improved.

Furthermore, we also calculate the values of *Q*_*MI*_, *Q*_*TE*_, *Q*_*NCIE*_, *Q*_*G*_, *Q*_*P*_, *Q*_*S*_, *Q*_*Y*_, *Q*_*CV*_, and *Q*_*CB*_ of the fused images obtained by DSIFT algorithm on the dataset before and after standardization of the image basic attribute. The calculated results of the nine metrics are shown in Table 3, respectively. From which one can find that in addition to the decrease of the value, the values of *Q*_*MI*_, *Q*_*TE*_, *Q*_*NCIE*_, *Q*_*G*_, *Q*_*P*_, *Q*_*S*_, *Q*_*Y*_, and *Q*_*CB*_ of the fused images obtained by the DSIFT algorithm on the dataset after the standardization of the image basic attribute are all increased. Therefore, after the dataset is standardized by the image basic attribute, both the subjective vision and the objective evaluation are all improved.

**Table 3:** The nine metrics' values of the fused images before and after standardization of the image basic attribute.

**Test image**	**Preprocessing**	***Q*_*MI*_**	***Q*_*TE*_**	***Q*_*NCIE*_**	***Q*_*G*_**	***Q*_*P*_**	***Q*_*S*_**	***Q*_*Y*_**	***Q*_*CV*_**	***Q*_*CB*_**
Blue black box	Before normalization	1.049	0.4295	0.8333	0.597	0.6621	0.8845	0.8211	156.4	0.6423
image	After normalization	1.151	0.4546	0.8372	0.6216	0.738	0.9515	0.9012	13.72	0.6541
White black box	Before normalization	1.199	0.4643	0.8445	0.6224	0.5284	0.9153	0 8392	43.79	0.6182
image	After normalization	1.233	0.4664	0.8454	0.6274	0.5985	0.9559	0.8899	24.99	0.6196
Bottle cap image	Before normalization	1.037	0.4542	0.8219	0.6639	0.5057	0.8898	0.8412	398.5	0.5374
	After normalization	1.196	0.4557	0.8303	0.6966	0.5568	0.9808	0.9081	25.64	0.657

#### Test of existing image fusion algorithms

**Figure 5:**
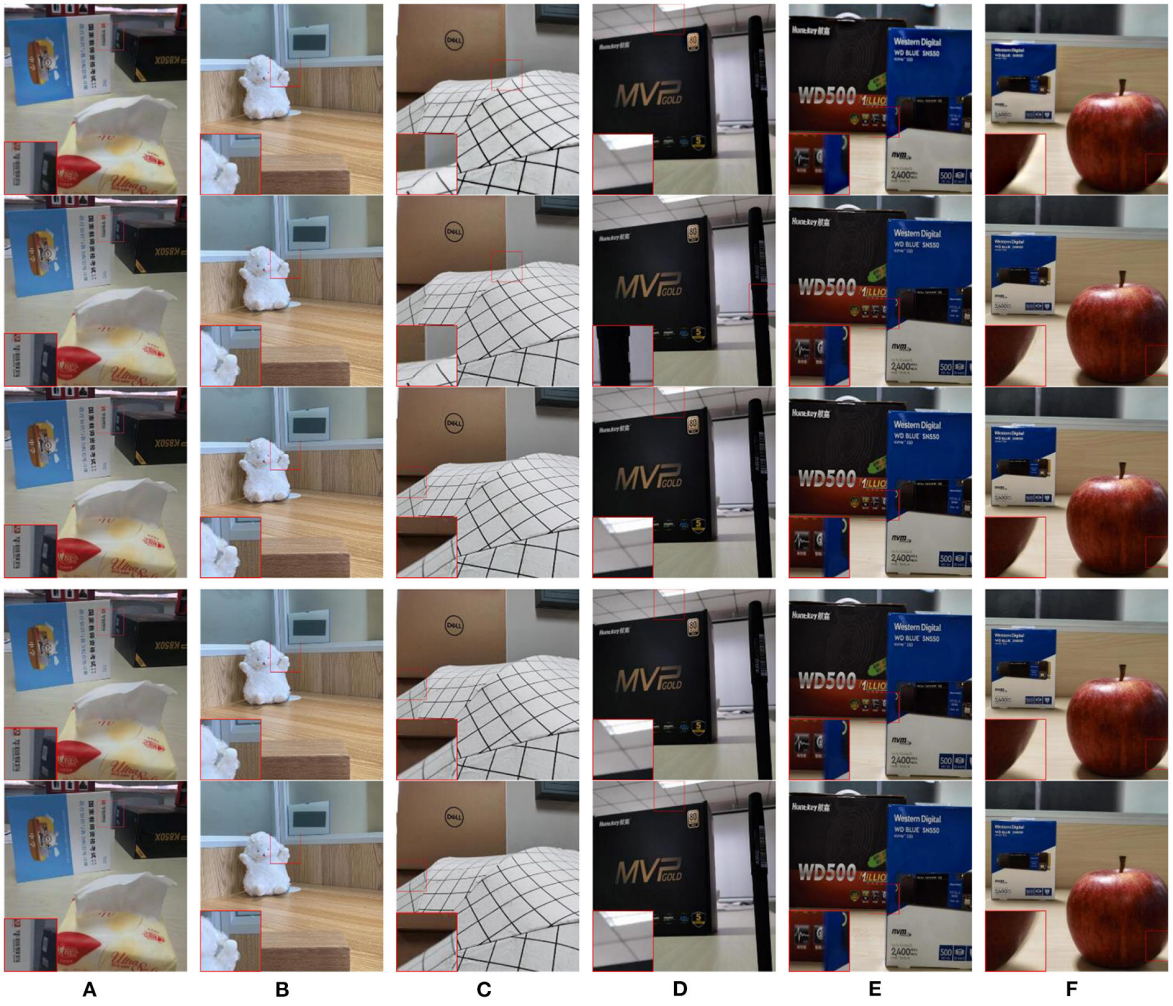
Fusion results of different algorithms. **(A–F)** are the fusion results of MGF, DSIFT, IFCNN, CNN, and SESF, respectively.

In this subsection, we test the performance of some existing image fusion algorithms on the HBU-CVMDSP dataset. The multi-focus image fusion algorithms used in the test include multi-scale guided filtering algorithm (MGF) (Bavirisetti et al., 2019), dense scale-invariant feature transformation algorithm (DSIFT) (Liu et al., 2015), a general image fusion algorithm based on convolutional neural network (IFCNN) (Zhang et al., 2020), MIF algorithm based on convolutional neural network (CNN) (Liu et al., 2017), and unsupervised depth model for MIF (SESF) (Ma et al., 2020). We select six pairs of images from the HBU-CVMDSP dataset to test the above algorithms, and the selected images are shown in Figure 1. Figure 5 shows the fusion results of different algorithms on the selected images. In order to better show the visual effects of different fusion algorithms, the image of the red rectangular area in the figure is enlarged in this paper. From the Figure 5, it can be found that the fused image obtained all the fusion methods are all kinds of problems, such as block effect, unfocused pixels on the edge, blurred edges, the detailed information lost, the boundary too smooth, artificial artifacts, misclassification of focused pixels, distorted, and poor spatial consistency.

The nine metrics' values of the fused images in Figure 5 are shown in Table 4, in which the best result of each group of fused images is bolded. As can be seen from the Table 4, in the objective evaluation of the fusion results of MGF, DSIFT, IFCNN, CNN and SESF in the real environment, no fusion algorithm has competitive performance compared with other comparison algorithms, which indicates that the multi focus image dataset in the real environment can reflect that the existing fusion algorithms cannot meet the application of MIF technology in the real environment. In addition, due to the limited generalization ability, these existing fusion algorithms all transfer specific prior knowledge to the model, and then perform image fusion. However, images in the real world are very complex, and cannot be achieved only through the prior knowledge of inherent images. Therefore, the HBU-CVMDSP dataset can be used as a new test set to promote the development of the field of MIF and narrow the gap between the theoretical and real environmental data of image fusion algorithms.

**Table 4:** The nine metrics' values of the fused images obtained by different fusion algorithms.

**Fused images**	**Fusion algorithms**	***Q*_*MI*_**	***Q*_*TE*_**	***Q*_*NCIE*_**	***Q*_*G*_**	***Q*_*P*_**	***Q*_*S*_**	***Q*_*Y*_**	***Q*_*CV*_**	***Q*_*CB*_**
Figure 5A	MGF	0.9503	0.4118	0.8337	0.5144	0.6205	0.9703	0.8081	171.61	0.6218
	DSIFT	**1.331**	**0.4474**	**0.8591**	**0.6593**	0.7285	0.9713	0.9365	52.805	0.7087
	IFCNN	1.1692	0.4364	0.8449	0.5663	0.6786	**0.9751**	0.86	**36.691**	0.6591
	CNN	1.2944	0.4449	0.8548	0.6515	**0.7599**	0.9736	**0.9478**	52.857	**0.7094**
	SESF	1.2839	0.4412	0.8544	0.641	0.7201	0.9696	0.9438	52.823	0.7079
Figure 5B	MGF	1.0355	**0.4622**	0.8336	0.6567	0.7942	**0.9697**	0.9066	**7.5085**	0.7040
	DSIFT	1.259	0.4419	0.8457	0.7262	0.9103	0.9673	0.9595	8.991	0.6965
	IFCNN	1.0766	0.4535	0.8356	0.6711	0.8433	0.967	0.9241	9.2736	0.6853
	CNN	1.2673	0.4456	0.8471	0.7364	**0.9132**	0.9688	**0.9707**	9.4967	0.7087
	SESF	**1.2852**	0.4477	**0.8491**	**0.9319**	0.9118	0.9681	0.9705	9.3427	**0.7196**
Figure 5C	MGF	0.8845	0.3979	0.8284	0.5109	0.6787	0.9507	0.8332	131.73	0.6279
	DSIFT	**1.3773**	**0.455**	**0.8593**	0.696	0.7722	0.9646	0.9793	119.89	0.7871
	IFCNN	1.0918	0.4162	0.8381	0.5677	0.7317	0.9642	0.8996	**35.251**	0.6962
	CNN	1.3699	0.4519	0.8582	**0.6993**	**0.7732**	**0.9649**	**0.9898**	119.86	**0.7949**
	SESF	1.3498	0.4481	0.857	0.6913	0.7717	**0.9649**	0.9813	35.31	0.7902
Figure 5D	MGF	1.0282	0.4094	0.8352	0.5355	0.6556	0.9598	0.7767	41.591	0.6395
	DSIFT	**1.4076**	**0.4498**	**0.8602**	0.6732	0.792	0.9714	0.9287	46.321	0.7394
	IFCNN	1.2627	0.4372	0.8474	0.5987	0.7460	**0.9727**	0.8541	**36.335**	0.6912
	CNN	1.3808	0.4464	0.8561	**0.6804**	**0.8007**	0.9722	**0.9562**	44.702	**0.7575**
	SESF	1.3962	0.4472	0.859	0.673	0.7884	0.9707	0.9454	44.88	0.7504
Figure 5E	MGF	0.9027	0.412	0.8293	0.526	0.6821	0.9508	0.7965	72.736	0.6049
	DSIFT	**1.3144**	0.438	**0.8497**	**0.6772**	0.8211	0.9654	0.9442	**23.078**	0.7069
	IFCNN	1.1571	**0.4413**	0.8406	0.5843	0.7547	**0.9691**	0.8659	26.5	0.6623
	CNN	1.2906	0.4363	0.848	0.6771	**0.8369**	0.9675	**0.9659**	23.374	**0.7234**
	SESF	1.2784	0.4334	0.8474	0.6656	0.8083	0.9641	0.9424	29.576	0.7165
Figure 5F	MGF	0.9452	0.4101	0.8323	0.5279	0.5967	0.9611	0.8309	96.451	0.6142
	DSIFT	**1.32**	**0.4479**	**0.8531**	0.6927	0.7965	0.9666	0.9684	39.348	0.7202
	IFCNN	1.174	0.4388	0.8436	0.5964	0.7127	**0.9691**	0.9	**19.685**	0.6651
	CNN	1.3072	0.447	0.8522	**0.6949**	**0.8299**	0.9678	0.9776	38.345	**0.7232**
	SESF	1.297	0.4451	0.8521	0.6915	0.8053	0.9669	**0.9784**	45.9072	0.7208

The bold values represent optimal values.

## Conclusion

Due to the existing MIF datasets cannot reflect the image registration caused by physical movement or camera shake, and the brightness differences caused by illumination in real life, we proposed a new MIF dataset i.e., the HBU-CVMDSP dataset. Images in this dataset are captured by smartphone, and can truly reflect the real-world scene. In addition, we test the performance of some existing fusion algorithms on the proposed dataset. The results indicate that the performance of these algorithms on the proposed dataset has much room for improvement. Therefore, the HBU-CVMDSP dataset can better promote the research of the MIF algorithms.

## Data availability statement

The datasets presented in this study can be found in online repositories. The names of the repository/repositories and accession number(s) can be found below: https://www.researchgate.net/publication/359468841.

## Author contributions

WP and YY performed the computer simulations. SL, WJ, and JZ analyzed the data. SL, WP, and YY wrote the original draft. WJ, YS, and JZ revised and edited the manuscript. YS polished the manuscript. All authors confirmed the submitted version.

## Funding

This work was supported in part by National Natural Science Foundation of China under Grant No. 62172139, Natural Science Foundation of Hebei Province under Grant No. F2022201055, Project Funded by China Postdoctoral under Grant No. 2022M713361, Science Research Project of Hebei Province under Grant No. BJ2020030, Natural Science Interdisciplinary Research Program of Hebei University under Grant No. DXK202102, Open Project Program of the National Laboratory of Pattern Recognition (NLPR) under Grant No. 202200007, Foundation of President of Hebei University under Grant No. XZJJ201909, Research Project of Hebei University Intelligent Financial Application Technology R&D Center under Grant No. XGZJ2022022, and Open Foundation of Guangdong Key Laboratory of Digital Signal and Image Processing Technology under Grant No. 2020GDDSIPL-04.

## Conflict of interest

The authors declare that the research was conducted in the absence of any commercial or financial relationships that could be construed as a potential conflict of interest.

## Publisher's note

All claims expressed in this article are solely those of the authors and do not necessarily represent those of their affiliated organizations, or those of the publisher, the editors and the reviewers. Any product that may be evaluated in this article, or claim that may be made by its manufacturer, is not guaranteed or endorsed by the publisher.

## References

[B1] Bavirisetti D. P. Xiao G. Zhao J. Dhuli R. Liu G. (2019). Multi-scale Guided image and video fusion: a fast and efficient approach. Circ. Syst. Signal Proc. 38, 5576–5605. 10.1007/s00034-019-01131-z

[B2] Bouzos O. Andreadis I. Mitianoudis N. (2019). Conditional random field model for robust multi-focus image fusion. IEEE Trans. Image Proc. 28, 5636–5648. 10.1109/TIP.2019.2922097 31217116

[B3] Liu S. Ma J. Yang Y. Qiu T. Li H. Hu S. . (2022). A multi-focus color image fusion algorithm based on low vision image reconstruction and focused feature extraction. Signal Proc. Image Commun. 100, 116533. 10.1016/j.image.2021.116533

[B4] Liu S. Ma J. Yin L. Li H. Cong S. Ma X. . (2020). Multi-focus color image fusion algorithm based on super-resolution reconstruction and focused area detection. IEEE Access 8, 90760–90778. 10.1109/ACCESS.2020.2993404

[B5] Liu S. Miao S. Su J. (2021). UMAG-Net: A new unsupervised multiattention-guided network for hyperspectral and multispectral image fusion. IEEE J. Select. Top. Appl. Earth Observat. Remote Sens. 14, 7373–7385. 10.1109/JSTARS.2021.3097178

[B6] Liu S. Wang J. Lu Y. Li H. Zhao J. Zhu Z. (2019). Multi-focus image fusion based on adaptive dual-channel spiking cortical model in non-subsampled shearlet domain. IEEE Access 7, 56367–56388. 10.1109/ACCESS.2019.2900376

[B7] Liu Y. Chen X. Peng H. Wang Z. (2017). Multi-focus image fusion with a deep convolutional neural network. Informat. Fusion 36, 191–207. 10.1016/j.inffus.2016.12.00132248098

[B8] Liu Y. Liu S. Wang Z. (2015). Multi-focus image fusion with dense SIFT. Inf. Fusion 23, 139–155. 10.1016/j.inffus.2014.05.004

[B9] Ma B. Zhu Y. Yin X. Ban X. Huang H. Mukeshimana M. (2020). SESF-Fuse: an unsupervised deep model for multi-focus image fusion. Neural Comput. Appl. 33, 5793–5804. 10.1007/s00521-020-05358-9

[B10] Nejati M. Samavi S. Shirani S. (2015). Multi-focus image fusion using dictionary-based sparse representation. Inf. Fusion 25, 72–84. 10.1016/j.inffus.2014.10.004

[B11] Su X. Li J. Hua Z. (2022). Transformer-based regression network for pansharpening remote sensing images. IEEE Trans. Geosci. Remote Sens. 60, 5407423. 10.1109/TGRS.2022.3152425

[B12] Willenbockel V. Sadr J. Fiset D. Horne G. O. Gosselin F. Tanaka J. W. (2010). Controlling low-level image properties: the SHINE toolbox. Behav. Res. Methods 42, 671–684. 10.3758/BRM.42.3.671 20805589

[B13] Xiao B. Ou G. Tang H. Bi X. Li W. (2020). Multi-focus image fusion by hessian matrix based decomposition. IEEE Trans. Multimedia 22, 285–297. 10.1109/TMM.2019.2928516

[B14] Zhang Y. Liu Y. Sun P. Yan H. Zhao X. Zhang L. (2020). IFCNN: a general image fusion framework based on convolutional neural network. Inf. Fusion 54, 99–118. 10.1016/j.inffus.2019.07.011

[B15] Zheng M. Qi G. Zhu Z. Li Y. Wei H. Liu Y. (2020). Image dehazing by an artificial image fusion method based on adaptive structure decomposition. IEEE Sens. J. 20, 8062–8072. 10.1109/JSEN.2020.2981719

[B16] Zhu Z. Wei H. Hu G. Li Y. Qi G. Mazur N. (2021). A novel fast single image dehazing algorithm based on artificial multiexposure image fusion. IEEE Trans. Instrum. Meas. 70, 1–23. 10.1109/TIM.2020.302433533776080

